# A Comparison of Diarrheal Severity Scores in the MAL-ED Multisite Community-Based Cohort Study

**DOI:** 10.1097/MPG.0000000000001286

**Published:** 2016-10-24

**Authors:** Gwenyth O. Lee, Stephanie A. Richard, Gagandeep Kang, Eric R. Houpt, Jessica C. Seidman, Laura L. Pendergast, Zulfiqar A. Bhutta, Tahmeed Ahmed, Estomih R. Mduma, Aldo A. Lima, Pascal Bessong, Mats Steffi Jennifer, Md. Iqbal Hossain, Ram Krishna Chandyo, Emanuel Nyathi, Ila F. Lima, John Pascal, Sajid Soofi, Bodhidatta Ladaporn, Richard L. Guerrant, Laura E. Caulfield, Robert E. Black, Margaret N. Kosek

**Affiliations:** ∗ Johns Hopkins Bloomberg School of Public Health, Baltimore; † Fogarty International Center, National Institutes of Health, Bethesda, MD; ‡ Christian Medical College, Vellore, India; § University of Virginia, Charlottesville; || Temple University, Philadelphia, PA; ¶ Aga Khan University, Karachi, Pakistan; # icddr,b (formerly International Centre for Diarrhoeal Disease Research, Bangladesh), Dhaka, Bangladesh; ∗∗ Haydom Lutheran Hospital, Haydom, Manyara Region, Tanzania; †† Federal University of Ceará, Fortaleza, Brazil; ‡‡ University of Venda, Thohoyandou, South Africa; §§ Centre for International Health, University of Bergen, Bergen, Norway; |||| Department of Child Health, Institute of Medicine, Tribhuvan University, Kathmandu, Nepal; ¶¶ Armed Forces Research Institute of Medical Sciences, Bangkok, Thailand.

**Keywords:** diarrhea, epidemiology, pediatric

## Abstract

**Objectives:**

There is a lack of consensus on how to measure diarrheal severity. Within the context of a multisite, prospective cohort study, we evaluated the performance of a modified Vesikari score (MAL-ED), 2 previously published scores (Clark and CODA [a diarrheal severity score (Community DiarrheA) published by Lee et al]), and a modified definition of moderate-to-severe diarrhea (MSD) based on dysentery and health care worker diagnosed dehydration.

**Methods:**

Scores were built using maternally reported symptoms or fieldworker-reported clinical signs obtained during the first 7 days of a diarrheal episode. The association between these and the risk of hospitalization were tested using receiver operating characteristic analysis. Severity scores were also related to illness etiology, and the likelihood of the episode subsequently becoming prolonged or persistent.

**Results:**

Of 10,159 episodes from 1681 children, 143 (4.0%) resulted in hospitalization. The area under the curve of each score as a predictor of hospitalization was 0.84 (95% confidence interval: 0.81, 0.87) (Clark), 0.85 (0.82, 0.88) (MAL-ED), and 0.87 (0.84, 0.89) (CODA). Severity was also associated with etiology and episode duration. Although families were more likely to seek care for severe diarrhea, approximately half of severe cases never reached the health system.

**Conclusions:**

Community-based diarrheal severity scores are predictive of relevant child health outcomes. Because they require no assumptions about health care access or utilization, they are useful in refining estimates of the burden of diarrheal disease, in estimating the effect of disease control interventions, and in triaging children for referral in low- and middle-income countries in which the rates of morbidity and mortality after diarrhea remain high.

## Abstract 2

Supplemental Digital Content is available in the text

**What Is Known**

- There is a lack of consensus on how to define severity in community-based studies of childhood diarrhea in the low- and middle-income countries.
- This lack of consensus limits comparability between studies.
- Existing severity scores were developed around rotavirus, and their reliability and validity in relation to non-rotavirus diarrhea is unknown.

**What Is New**

- Severity scores built using maternally reported symptoms predicted hospitalization and other negative child health outcomes across geographically diverse communities.
- Validated clinical severity criteria can be used to quantify estimates of severe diarrhea in the absence of outpatient confirmation.
- The global health research community should adopt standardized methods of measuring diarrheal severity

The lack of validated and widely adopted definitions for severity is a methodological hindrance to community-based and outpatient studies of pediatric diarrhea in low- and middle-income countries (1). A recent review highlighted heterogeneity in measurement approaches; among 138 randomized clinical trials involving primary outcomes of acute diarrhea, 46 definitions were used, and in 32 trials using diarrheal severity instruments, 8 used the original or modified Vesikari score (2–4), whereas the remainder used other instruments. This review also noted that there are no published reports of reliability and validity for any diarrheal severity instrument (5).

The Vesikari score was designed for use in rotavirus vaccine trials (6), and although useful in that context (7), it may be less so in community-based studies due to multiple etiologies. This is because it includes clinical management in its scoring, making it impractical in contexts in which diarrhea referral and treatment varies, weighs vomiting relatively heavily compared with other symptoms, and does not include dysentery (8). The original Vesikari score assessed children at hospital presentation (9), but subsequent studies have calculated severity after episode resolution (10–12). Other scores have been proposed (13,14), but only 1 designed for nonetiologically specific diarrhea in HIV-negative children (15).

Conceptually, diarrheal severity relates to acute morbidity, represented by the presence of clinical signs and symptoms such as fever or vomiting; the risk of acute progression, including severe dehydration, and, in the worst case, mortality; and the risk of later morbidity (eg, nutritional status) (15). Although these domains overlap, there may be differences in which should be targeted during the clinical assessment of severity in an outpatient context, versus during community surveillance where the majority of episodes may be mild-to-moderate. Although different scores may serve for different purposes, the agreement between them should nevertheless be characterized.

The Etiology, Risk Factors, and Interactions of Enteric Infections and Malnutrition and the Consequences for Child Health and Development (MAL-ED) study is an 8-site, longitudinal cohort designed to investigate effects of enteric infection and dietary intake on child growth and development (16). Here, we describe a modified Vesikari score constructed for MAL-ED, 2 previously published scores (2,15), and a definition of moderate-to-severe diarrhea (MSD) defined as dysentery, dehydration, or hospitalization, based on the case inclusion criteria in a recent multisite study of diarrheal etiology (17,18). We evaluated score validity by comparing against the risk of hospitalization and the risk of an episode becoming prolonged or persistent, and illness etiology.

## PATIENTS AND METHODS

The MAL-ED study has been described elsewhere (16). Briefly, 8 sites (Dhaka, Bangladesh, Fortaleza, Brazil, Vellore, India, Bhaktapur, Nepal, Loreto, Peru, Naushero Feroze, Pakistan, Venda, South Africa, Haydom, Tanzania) used a harmonized protocol to each follow approximately 200 mother-newborn pairs for the first 2 years of life. Ethical approval from the institutional review boards at each of the participating research sites was obtained.

Study personnel visited each household biweekly using structured questionnaires to collect child symptom histories, based on caregiver report, for each of the 1 to 4 days prior: count of loose (semiliquid or liquid) stools, activity level (normal, sleepy, or difficult to awaken), oral intake (normal or more than normal vs less than normal), vomiting, fever, bloody stool, and dehydration (none, some [irritable, thirsty, delay in skin pinch, sunken eyes], or severe [symptoms more severe with lethargy and listlessness]). Fieldworkers were trained to use the World Health Organization (WHO-IMCI) dehydration scale (19) and they in turn trained caregivers to recognize dehydration (20).

Care seeking for the child was reported on a referral form with structured fields for: the reason for seeking care (diarrhea/acute lower respiratory infection/malaria/growth/anemia/other), whether study personnel prompted the care seeking, location of care (pharmacy/health center/other), the diagnosis resulting from the visit (dehydrating diarrhea, ALRI, and/or malaria), and hospitalization. Per protocol, study staff directed families to seek care whenever ≥8 semiliquid or liquid stools in a 24-hour period, or bloody stools, were reported.

Diarrheal stool samples were collected and tested for a broad panel of viral, protozoal, and bacterial enteropathogens (21).

### Definitions

MAL-ED defined diarrheal episodes as ≥3 loose stools per 24-hour period, or ≥1 loose stool with blood, separated by ≥2 days not meeting this definition (20,22). Instances in which a lactulose: mannitol urine test occurred that day or the previous day were excluded, because lactulose is an osmotic laxative. All episodes that occurred in the first 24 months of life were included, regardless of a child's total follow-up time.

Episodes were considered associated with care seeking whenever a referral completed during or 1 day after an episode indicated diarrhea as the primary reason for the visit, regardless of the location visited and whether study staff prompted the visit. Episodes associated with multiple referrals were classified as dehydrating diarrhea if any referral form indicated that diagnosis. When visits resulted in a dehydrating diarrhea diagnosis or hospitalization for diarrhea, then diarrhea was assumed as the reason for care-seeking, regardless of other symptoms indicated on the referral form.

“Hospitalization for diarrhea” was considered a proxy for severe diarrhea that was comparable across sites; referral form text fields were checked to confirm the reason for hospitalization. All hospitalizations associated with dehydrating diarrhea diagnoses, all hospitalizations following care-seeking for diarrhea not associated with any other diagnosis, and 5 episodes from India in which care-seeking reason was specified as “other” and there was no dehydrating diarrhea diagnosis, but the text field nevertheless suggested diarrhea, were included.

### Severity Scores

Three severity scores were considered (Table 1, Supplemental Digital Content 1, Table 1).

**TABLE 1:** Scoring of the Clark, MAL-ED, and modified CODA score

Score component	Modified Clark (2)	MAL-ED	Modified CODA (15)	Scoring
	Nonspecific			
Duration of diarrhea	1–4 Days	2–4 Days		1
	5–7 Days	5–7 Days		2
	≥8 Days	≥8		3
Max number of loose stools/day	2–4	<5	4–5	1
	5–7	5–7	6–7	2
	≥8	≥8	≥8	3
	Vomiting			
Duration of vomiting, days	2 Days	1 Day	1–2 Days	1
	3–5 Days	2 Days	3–4 Days	2
	≥6 Days	≥3 Days	≥5 Days	3
	Fever			
Duration of reported fever, days	1–2 Days	Any	1–2 Days	1
	3–4 Days	−	3–4 Days	2
	≥5 Days	−	≥5 days	3
Confirmed temperature	38.0–38.2°C	−	−	1
	38.3–38.7°C	≥37.5°C (confirmed)	−	2
	38.8°C	−	−	3
	Dehydration/liquid stools			
Dehydration	−	−	1–2 Days	1
	−	Some dehydration reported	3–4 Days	2
	−	Severe dehydration reported	≥5 Days	3
	Behavioral signs			
Behavioral signs	Sleepy	−	−	1
	Difficult to awaken	−	−	2
	−	−	−	3
Behavioral signs (duration)	1–2 Days	−	−	1
	3–4 Days	−	−	2
	≥5 Days	−	−	3
Anorexia	−	−	1–2 Days	1
	−	−	3–4 Days	2
	−	−	≥5 Days	3
Total	23 Points	14 Points	15 Points	

First, a modified Vesikari score developed within the present study (MAL-ED) (8), the distinctive feature being that fieldworker-confirmed fever (axillary temperature ≥37.5°C) was scored more highly than maternally reported fever alone.

Secondly, the modified Vesikari score developed by Clark and colleagues (2) was used, with further changes based on data collected: omission of the number of emeses per day, substitution of the number of loose stools/24 hours for the total number of stools/24 hours, and behavioral symptoms irritable = 1, lethargic/listless = 2, and seizures = 3 replaced with sleepy = 1 and difficult to awaken = 2.

Finally, the “CODA score (a diarrheal severity score [Community Diarrhea] published by Lee et al)” (15) was modified based on available data: total loose stools/24 hours replaced the total number of stools/24 hours, the number of days with ≥4 liquid stools reported was excluded, and the number of days with caregiver-reported dehydrated was added.

All acute (≤7 days), prolonged (8–13 days), and persistent (≥14 days) diarrheal episodes were included. To maximize comparability with other studies and to compare with the outcome of prolonged/persistent diarrhea (17), scores were calculated using only symptoms reported within the first 7 days of onset, limiting Clark and MAL-ED scales to 22 and 13 point maximums, respectively.

We did not consider other instruments such as the original Vesikari (9) and Freedman et al scores (3) because they include components reflecting clinical decision-making (eg, outpatient vs emergency treatment), which we wished to test as outcomes of severity rather than components thereof.

### Moderate-to-Severe Diarrhea

When care-seeking occurred, we compared each of the 3 severity scores to MSD (ie, CODA score to MSD; MAL-ED score to MSD; Clark score to MSD), that is, dehydrating diarrhea diagnosed by a health care worker (HCW), caregiver-reported visible fecal blood, or hospitalization for diarrhea. Caregiver-reported dehydration was not considered because we wished to define MSD independent of caregiver-report-derived severity scores. Agreement between caregiver-reported and HCW-reported dehydration was assessed using kappa statistics and logistic regression.

HCW dysentery diagnoses were captured from 3 sites (Bangladesh, Nepal, and Peru) by searching referral form text fields for terms such as “dysentery” and “invasive diarrhea” (Supplemental Digital Content 2, Appendix 1).

## STATISTICAL METHODS

### Performance

We used receiver operating characteristics (ROC) analysis to compare the area under the curve (AUC) of each score to the hospitalization for diarrhea outcome; the cut-off for each score was chosen as the value at which the sum of sensitivity and specificity was maximized (23). Differences between ROC curves were tested using 2-sided hypothesis tests of AUC equality.

Mean scores were calculated for episodes for which care was sought, dehydrating diarrhea was diagnosed, and hospitalization. Associations between individual score components and each outcomes were modeled with logistic regression that included categorical adjustment variables for study site, child age (<3, 3–6, 6–12, 12–18, and 18–24 months), and a child-level random intercept to account for children contributing multiple episodes to the analysis.

### Prolonged and Persistent Diarrhea

Associations between score components, overall scores, and the likelihood of an episode becoming prolonged/persistent were tested using logistic regression models with a child-level random intercept and adjustments for site and age. Overall scores were compared using 2-sided *t* tests and ROC analysis. ROC curves were compared by age and between sites except Brazil and South Africa, where prolonged/persistent episodes were rare. The MAL-ED and Clark scores were also tested with episode duration excluded from the score.

Throughout, sensitivity analyses were performed to confirm that prolonged and persistent episodes were not driving results, and treating prolonged and persistent as separate categories, rather than combined.

### Etiology

Regression models were built with the outcome of the episode severity and the predictors as the presence/absence of specific enteropathogens in stools collected during or up to 1 day after the episode. Only enteropathogens detected during at least 100 episodes (adenovirus, aeromonas, astrovirus, atypical and typical enteropathogenic *Escherichia coli*, enteroaggregative *E coli*, enteroinvasive *E coli*, heat-stable (ST)- and heat-lable (LT)-enterotoxigenic *E coli*, *Campylobacter*, *Cryptosporidium*, *Giardia*, norovirus GI, norovirus GII, rotavirus, and *Shigella*) were considered. Models included a child-level random intercept and adjusted for site, but not age. Based on sensitivity analyses, prolonged/persistent episodes were excluded.

All *P* value of ≤0.05 were regarded as “statistically significant.” Analysis were done using Stata version 12.1 (College Station, TX).

## RESULTS

Of 2145 children enrolled, 1681 experienced 10,159 diarrheal episodes including 924 (9.1%) prolonged and 351 (3.5%) persistent episodes. Nineteen children died and diarrhea was implicated in 5 deaths: 2 also had respiratory infections (Pakistan), 1 with galactosemia (India), and 2 with diarrhea and fever without other symptoms (Pakistan and Bangladesh).

There were 143 hospitalizations associated with 107 acute, 28 prolonged, and 8 persistent episodes (Table 2).

**TABLE 2:** Episodes of diarrhea summarized

Site	Children	Total episodes	Prolonged	Persistent	Blood in stool by maternal report	Cases referred due to diarrhea^*^	Diagnosis of dehydrating diarrhea	MSD^†^	Hospitalization due to diarrhea
BGD	242	1670	106	22	70	985	74	140	51
BRF	100	180	14	1	4	58	2	5	0
INV	218	974	49	14	59	360	14	59	20
NEP	221	1077	149	34	49	382	38	77	23
PEL	270	2102	175	39	110	632	238	296	16
PKN	271	3210	414	241	90	809	188	217	6
SAV	154	323	6	0	11	55	9	15	6
TZH	205	623	11	0	84	297	219	226	21
Total	1681	10,159	924	351	477	3578	773	1035	143

MSD = moderate-to-severe diarrhea.

^*^Diarrhea as the primary reason for care-seeking, or care-seeking for any reason that resulted in a diagnosis of severe (dehydrating or dysenteric) diarrhea.

^†^Diagnosis of dehydrating diarrhea, maternally reported blood in stool, and/or hospitalization due to diarrhea (among episodes first referred for care only).

Health care was sought for 3578 episodes (35.2%) including 1035 classified as MSD: 590 with HCW-diagnosed dehydrating diarrhea, 213 with caregiver-reported blood, 46 with hospitalization, 89 with dehydration and reported blood, 87 with dehydration and hospitalization, 3 with hospitalization and reported blood, and 7 episodes with all 3 indicators.

### Individual Symptoms and Agreement Between Severity Measures

There were differences in reported symptoms between study sites (Table 3).

**TABLE 3:** Prevalence of symptoms reported by site

Site	BGD	BRF	INV	NEB	PEL	PKN	SAV	TZH
Anorexia	35.8%	7.8%	20.6%	2.7%	34.9%	2.7%	22.6%	50.9%
Dysentery^*^	4.2%	2.2%	6.1%	4.6%	5.2%	2.8%	3.7%	13.5%
Fever	22.8%	15.6%	25.9%	27.9%	22.7%	46.2%	13.6%	24.6%
Reduced activity level	3.1%	1.1%	11.2%	3.3%	14.5%	0.3%	19.5%	46.6%
Vomiting	30.1%	27.8%	22.0%	18.8%	18.6%	31.3%	19.5%	37.1%
Dehydration^†^	4.4%	0.6%	4.0%	4.3%	9.4%	15.6%	3.4%	6.4%
Axillary temperature ≥37.5 (n = 1368)	21.4%	78.6%	9.6%	23.8%	33.9%	11.2%	31.6%	8.1%
Median MLS (10th, 90th per)	5 (3, 8)	4 (3, 5.5)	5 (3, 10)	6 (4, 10)	5 (3, 7)	5 (3, 8)	4 (3, 6)	4 (3, 6)
Median duration (10th, 90th per)	3 (1, 7)	4 (2, 7)	2 (1, 7)	4 (2, 9)	3 (1, 8)	4 (1, 11)	2 (1, 4)	2 (1, 5)
Mean Clark score	3.7	3.2	3.7	4.3	3.7	4.3	3.1	4.3
Mean MAL-ED score	3.6	3.3	3.1	4.1	3.4	4.0	2.4	3.1
Mean CODA score	2.9	1.5	2.3	2.6	2.4	2.9	1.5	2.6

N = 10,159 for all values except axillary temperature; which is only reported for episodes, which at least 1 fieldworker-confirmed temperature measured during the course of the episode. Anorexia, dysentery, fever, reduced activity, vomiting, and dehydration are reported as the proportion of episodes in which the symptom was reported on at least 1 day, versus none.

^*^By maternal report.

^†^Some/severe by maternal report.

The Clark and MAL-ED scores were most highly correlated (Spearman's *ρ* = 0.84), followed by CODA and Clark (*ρ* = 0.83) and MAL-ED and CODA (*ρ* = 0.79). Among episodes in which care was sought, mean severity scores for MSD versus non-MSD was 4.3 versus 5.5 (Clark, a 5.4% difference), 4.0 versus 5.2 (MAL-ED, a 9.2% difference), and 3.1 versus 4.4 (CODA, an 8.7% difference) (*P* < 0.0001 for all). The AUC for all 3 scores predicting MSD was 0.65 (95% confidence interval [CI]: 0.63, 0.67).

### Hospitalization

**FIGURE 1:**
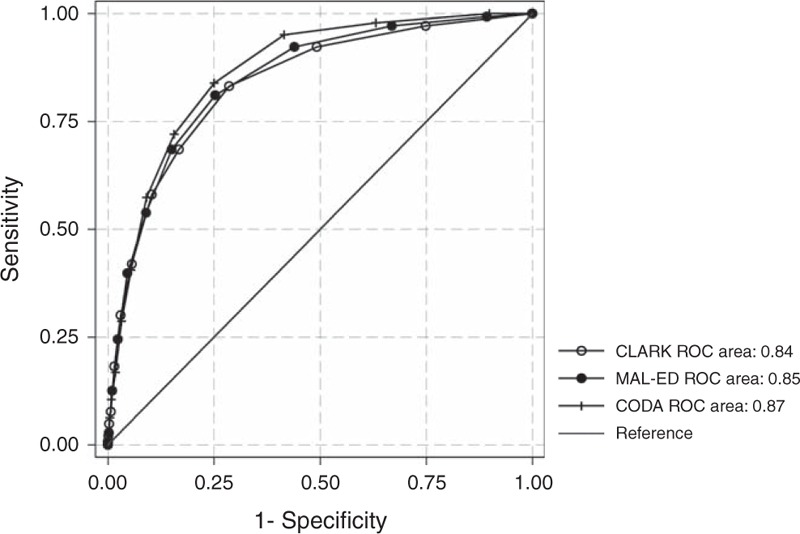
ROC curves for MAL-ED, CODA, and Clark scores. ROC = receiver operating characteristics.

The AUC of each scale predicting hospitalization was 0.84 (95% CI: 0.81, 0.87) (Clark), 0.85 (0.82, 0.88) (MAL-ED), and 0.87 (0.84, 0.89) (CODA) (Fig. 1, Supplemental Digital Content 1, Table 2). Overall, CODA was statistically significantly more predictive of hospitalization than did Clark (*P* = 0.0030), whereas the differences between CODA and MAL-ED, and Clark and MAL-ED, were not significant (*P* = 0.0610 and *P* = 0.3438, respectively). The best cut-off values were ≥5 for Clark (Se = 83.2%, Sp = 71.5%), ≥5 for MAL-ED (Se = 81.1%, Sp = 74.7%), and ≥4 for CODA (Se = 83.9%, Sp = 75.1%). Among episodes in which care was sought, AUCs fell to 0.78 (95% CI: 0.74, 0.82), 0.79 (95% CI: 0.75, 0.83), and 0.80 (95% CI: 0.77, 0.83), respectively (as a large number of never referred and therefore never eligible to be hospitalized cases were removed from consideration). Sensitivities were unchanged (all hospitalized cases were first referred) and specificities dropped to 58.6% (Clark), 61.8% (MAL-ED), and 61.9% (CODA).

All 3 scores were more predictive of hospitalization than any symptom individually (total loose stools/24 hours, dehydration, fever, anorexia, vomiting, and decreased activity level AUCs = 0.62–0.76; maternally reported dysentery AUC = 0.51, caregiver-reported dysentery and/or dehydration combined AUC = 0.63), or, among episodes in which care was sought, caregiver-reported dysentery, and/or HCW-diagnosed dehydration combined (AUC = 0.71). There were no statistically significant differences by age.

The fraction of episodes classified as severe was similar for each score: 29.3% (Clark ≥5), 26.1% (MAL-ED ≥5), and 25.9% (CODA ≥4). Care was sought for 51.7% of severe Clark episodes, 53.9% of severe MAL-ED episodes (45.5% in Pakistan to 69.5% in Bangladesh), and 54.4% of severe CODA episodes (45.4% in Pakistan to 72.7% in Brazil). Ignoring other symptoms, 12.8% of all episodes had caregiver-reported blood or dehydration, and among these, 61.9% sought care.

### Prolonged and Persistent Diarrhea

Prolonged/persistent episodes were associated with dehydration (odds ratio [OR] = 2.10, 95% CI: 1.72, 2.56) and fever (OR = 1.52, 95% CI: 1.32, 1.75) in the first 7 days, but not dysentery, vomiting, anorexia, or lethargy. Episodes that subsequently became prolonged/persistent were associated with higher scores than acute episodes (mean scores: MAL-ED = 5.5 vs 3.3, CODA = 4.0 vs 2.4, Clark = 5.7 vs 3.7), and scores were predictive of episodes becoming prolonged/persistent: MAL-ED AUC = 0.81 (95% CI: 0.80, 0.82); Clark AUC = 0.79 (95% CI: 0.78, 0.80), and CODA AUC = 0.70, (95% CI: 0.68, 0.71). When episode duration was excluded from scoring, AUCs were similar for all 3 scores (AUC = 0.70 for all). Statistically significant differences in AUCs both by age (eg, MAL-ED AUCs of 0.82 at ≤7 months, 0.79 at 7–12 months, and 0.80 at >12 months) and site (MAL-ED AUCs of 0.73–0.87) suggest variability in the predictive value of these scores attributable to these factors.

### Care-Seeking

The strongest individual predictors of care-seeking during an episode were dehydration (OR = 4.14, 95% CI: 3.48, 4.93) and dysentery (OR = 3.63, 95% CI: 3.67, 4.60). Other symptoms (lethargy, fever, anorexia, vomiting) were also significant (OR 1.39–1.55).

**FIGURE 2:**
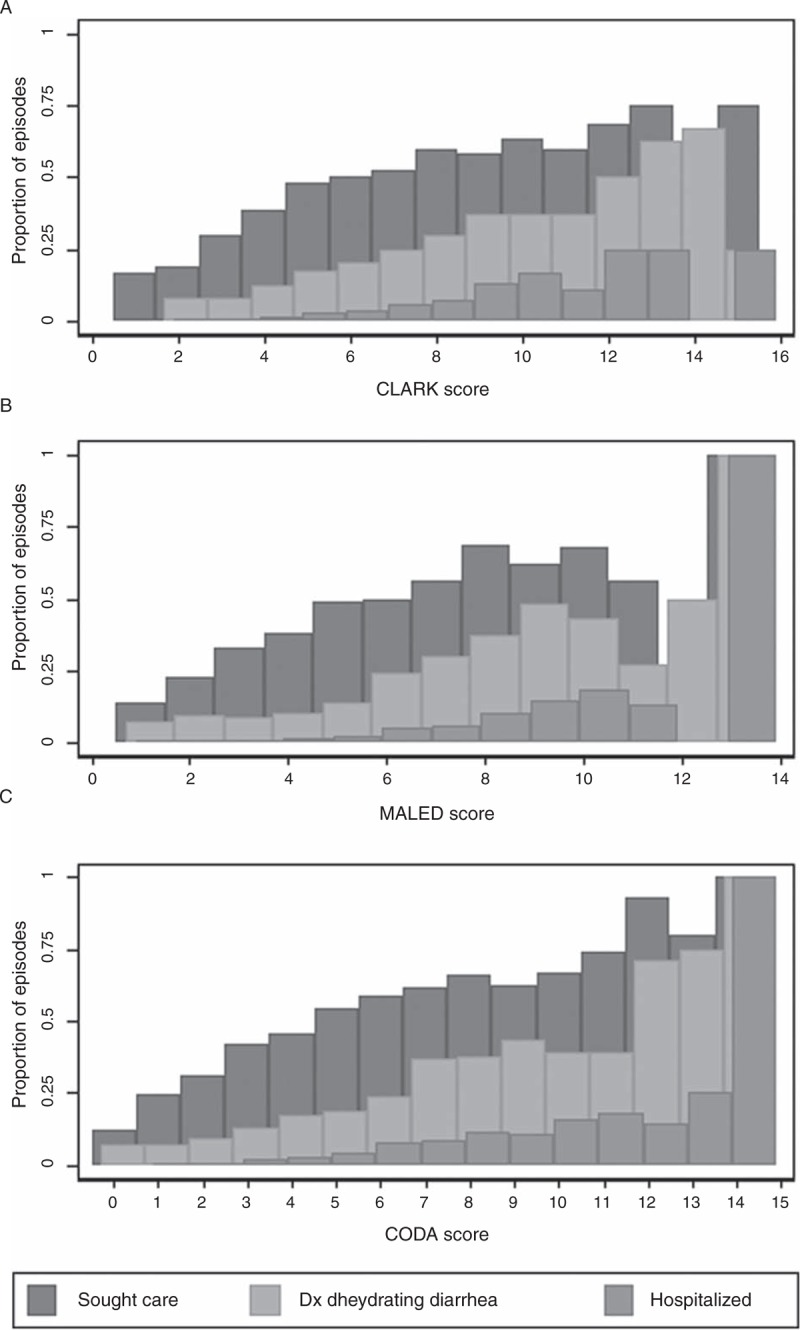
The proportion of episodes of any given score in which care was sought for the episode, care was sought and the doctor diagnosed dehydrating diarrhea, or the child was hospitalized. Only 10 episodes occurred with a Clark score of ≥14, 5 episodes had an MAL-ED score of 12 or 13, and 6 episodes had a CODA score of 13 or 14.

Mean episode severity scores based on care-seeking, diagnosis, and hospitalization are shown in Figure 2. Among episodes in which care was sought, maternally reported dehydration was the strongest predictor of HCW-reported dehydrating diarrhea (OR = 11.97, 95% CI: 8.77, 16.36, Cohen kappa = 0.37); vomiting and lethargy, but not fever, anorexia, or maternally reported dysentery, were also associated with receiving such a diagnosis (OR = 1.54, 95% CI: 1.19, 1.99 and OR = 2.10, 95% CI: 1.47, 3.02).

### Etiology

**FIGURE 3:**
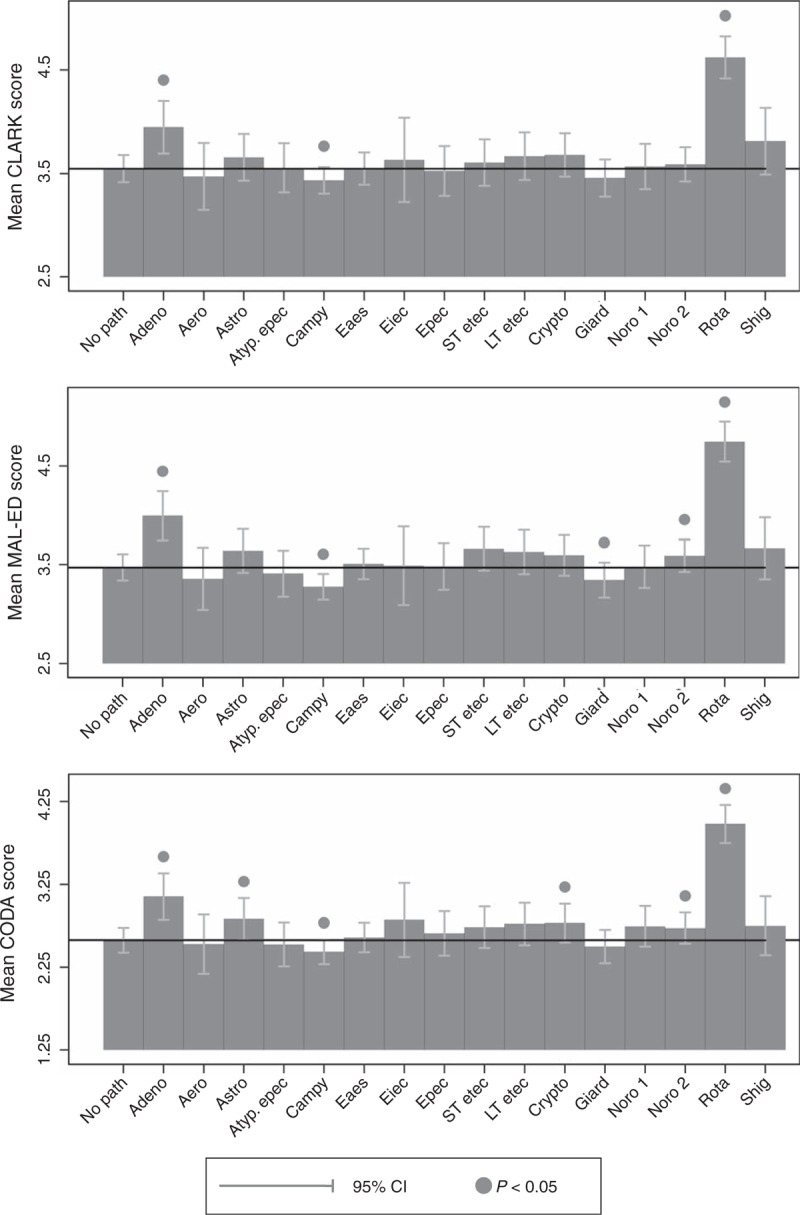
Bars reflect the mean score for diarrheal episodes associated with common study enteropathogens, and brackets reflect 95% confidence intervals, estimated from linear regressions, which adjusted for study site but not child age and included a random intercept to account for multiple episodes per child.

Among the commonly detected pathogens, adenovirus- and rotavirus-positive diarrhea were associated with higher severity, and *Campylobacter* with lower severity, by all 3 scores (Fig. 3). Norovirus GII were associated with greater severity by CODA and MAL-ED but not by Clark, astrovirus and *Cryptosporidium* with higher severity by CODA but not MAL-ED or Clark, and *Giardia* with lower severity by MAL-ED but not CODA or Clark.

## DISCUSSION

In this diverse multisite study, diarrhea severity scores based on twice-weekly caregiver-reported symptoms were associated with greater likelihood of hospitalization, and subsequent prolonged or persistent disease. Although other scores (ie, Vesikari) may be more appropriate when studying etiology-specific disease, we recommend using either MAL-ED or CODA score combined with caregiver-reported dysentery to measure non-etiologically specific childhood diarrheal severity at the community level.

Our analyses highlight the need to compare across studies using validated clinical severity criteria and to relate maternally reported severity to severity characterized in ambulatory settings. Elevated severity scores were associated with MSD defined as dehydrating diarrhea diagnoses, hospitalization, or maternally reported fecal blood; however, our finding that fecal blood was not associated with hospitalization suggests that using this criterion to identify severe episodes may be problematic.

Each score had limitations. The Clark score was previously found to agree poorly with the Vesikari (6,10,24,25). Higher Clark scores were positively associated with rotavirus detection while being relatively invariant to other enteropathogens. The MAL-ED score weighs fieldworker-confirmed fever more highly than maternally reported fever, but because many fevers occur outside of fieldworker visits, relatively few were confirmed. The CODA score includes reported anorexia, a symptom reported variably between sites, whereas more tangible signs such as fever and vomiting were reported more consistently.

Study personnel taught caregivers to recognize dehydration. Maternally reported dehydration appeared more meaningful than dehydration counted only on fieldworker visit days, likely because mothers had more observation time. Maternally reported dehydration was strongly associated with HCW-determined dehydration, consistent with the findings of other studies (17). Community programs to teach dehydration signs should be strengthened to ensure correct treatment of severe episodes, or, alternatively, potential signs of dehydration that can be readily identified without prior maternal training (such as the number of fully liquid stooling events) should be validated. One limitation is that “dehydration” was assessed without objective evidence such as the need for IV rehydration.

Because communication naturally occurs between caregiver, fieldworker, and clinician during diarrheal episodes, caregiver-reported severity is not independent of hospitalization. Caregiver-identified symptoms may have been reported after hospitalization which could affect recall.

Estimates of the global burden of enteric disease often use care-seeking as a surrogate of diarrhea severity (1). Our results suggest that although care-seeking increased with reported severity, there were also a substantial number of severe episodes according to maternally reported symptoms, in which no care was sought. Using an MAL-ED cut-off of ≥6, or a CODA cut-off of ≥5, approximately a quarter of diarrheal episodes in the community was classified as severe. Families sought care for only about half of these cases, similar to other estimates (1,26).

Diarrhea severity scores from caregiver-reported symptoms are valid, as they are able to predict relevant child health outcomes. Because these scores do not assume health care utilization or access, they should be incorporated into standardized definitions of diarrheal severity to refine estimates of total and enteropathogen-specific disease burdens and to assess the effect of disease control interventions.

## Supplementary Material

Supplemental Digital Content

## Supplementary Material

Supplemental Digital Content

## Supplementary Material

Supplemental Digital Content
