# Supplementary material for: A Comparison of Diarrheal Severity Scores in the MAL-ED Multisite Community-Based Cohort Study
Source: J Pediatr Gastroenterol Nutr. 2016 Oct 24;63(5):466–73. doi: 10.1097/MPG.0000000000001286 (PMC5084640; doi:10.1097/MPG.0000000000001286)
Supplement: Supplemental Digital Content [file jpga-63-466-s001.docx]

**Supplemental Table 1: Other common published scoring systems**

| **Direct Observation (DO) or caregiver Report (CR)** | **Score component** | **Vesakari** [9] | **Clark** [2] | **Freedman** [3] | **MAL-ED** | **CODA**[15] | **Scoring** |
| --- | --- | --- | --- | --- | --- | --- | --- |
|  |  | ***Non-specific*** | | | | | |
| **CR** | **Duration of Diarrhea** | 1-4 days | 1-4 days | 1-4 days | 2-4 days | - | 1 |
|  |  | 5 days | 5-7 days | 5 days | 5-7 days | - | 2 |
|  |  | ≥6 days | ≥8 days | ≥6 days | ≥8 days | - | 3 |
| **CR** | **Max number of stools /day** | 1-3 | 2-4 | 1-3 | <5 loose stools | 4-5 | 1 |
|  |  | 4-5 | 5-7 | 4-5 | 5-7 loose stools | 6-7 | 2 |
|  |  | ≥6 | ≥8 | ≥6 | ≥8 | ≥8 | 3 |
| **CR** | **Duration of ≥4 Liquid Stools (days)** | - | - | - |  | 1-2 days | 1 |
|  |  | - | - | - |  | 3-4 days | 2 |
|  |  | - | - | - |  | ≥5 days | 3 |
|  |  | ***Vomiting*** | | | | | |
| **CR** | **Duration of vomiting (days)** | 1 day | 2 days | 1 day | 1 day | 1-2 days | 1 |
|  |  | 2 days | 3-5 days | 2 days | 2 days | 3-4 days | 2 |
|  |  | ≥3 days | ≥6 days | ≥3 days | ≥3 days | ≥5 days | 3 |
| **CR** | **Number of emeses/day** | 1 | 1-3 | 1 | - | - | 1 |
|  |  | 2-4 | 4-6 | 2-4 | - | - | 2 |
|  |  | ≥5 | ≥7 | ≥5 | - | - | 3 |
|  |  | ***Fever*** | | | | | |
| **CR** | **Duration of fever (days)** | - | 1-2 days | - | Any | 1-2 days | 1 |
|  |  | - | 3-4 days | - | - | 3-4 days | 2 |
|  |  | - | ≥5 days | - | - | ≥5 days | 3 |
| **DO** | **Temperature / Rectal Temperature** | 37.1-38.4°C | 38.0-38.2°C | 37.1 – 38.4°C |  | - | 1 |
|  |  | 38.5-38.9°C | 38.3-38.7°C | 38.5 – 38.9°C | ≥37.5 | - | 2 |
|  |  | ≥ 39.0°C | 38.8°C | ≥39.0°C |  | - | 3 |
|  |  | ***Dehydration / Liquid Stools*** | | | | | |
| **DO** | **Dehydration** | - | - | - |  | - | 1 |
|  |  | 1-5% | - | - | any | - | 2 |
|  |  | ≥6% | - | - | severe | - | 3 |
|  |  | ***Behavioral Signs*** | | | | | |
| **CR** | **Behavioral signs** | - | irritable/less playful | **-** |  | **-** | 1 |
|  |  | - | lethargic/listless | **-** |  | **-** | 2 |
|  |  | - | seizures | **-** |  | **-** | 3 |
| **CR** | **Behavioral signs (duration)** | **-** | 1-2 days | - |  | - | 1 |
|  |  | **-** | 3-4 days | - |  | - | 2 |
|  |  | **-** | ≥5 days | - |  | - | 3 |
| **CR** | **Anorexia** | **-** | - | - |  | 1-2 days | 1 |
|  |  | **-** | - | - |  | 3-4 days | 2 |
|  |  | **-** | - | - |  | ≥5 days | 3 |
|  |  | ***Treatment*** | | | | | |
|  | **Treatment** | Rehydration | - | Rehydration |  | - | 1 |
|  |  | Hospitalization |  | Hospitalization |  | - | 2 |
|  | **Provider visit** |  | - | Outpatient |  | - | 2 |
|  |  |  |  | E.D. |  | - | 3 |
|  | **Total** | **20 points** | **24 points** |  |  | **15 points** |  |

**Supplemental Table 2: Severity Score agreement with Hospitalization due to diarrhea, by site**

(Hospitalization due to diarrhea defined as: Hospitalization + diagnosis of diarrhea reported in a single referral form).

| **Site** | **Clark Score Modified**  **AUC (95% CI)** | **MAL-ED Score**  **AUC (95% CI)** | **CODA Score Modified**  **AUC (95% CI)** |
| --- | --- | --- | --- |
| **BGD** | 0.85 (0.79, 0.90) | 0.87 (0.82, 0.91) | 0.84 (0.79, 0.89) |
| **BRF*** | - | - | - |
| **INV** | 0.86 (0.76, 0.95) | 0.82 (0.72, 0.93) | 0.88 (0.81, 0.95) |
| **NEB** | 0.84 (0.75, 0.92) | 0.91 (0.85, 0.97) | 0.91 (0.86, 0.97) |
| **PEL** | 0.90 (0.81, 0.98) | 0.91 (0.84, 0.97) | 0.92 (0.88, 0.96) |
| **PKN** | 0.76 (0.63, 0.90) | 0.90 (0.83, 0.97) | 0.90 (0.85. 0.95) |
| **SAV** | 0.92 (0.86, 0.99) | 0.95 (0.90, 1.00) | 0.94 (0.91, 0.98) |
| **TZH** | 0.85 (0.75, 0.94) | 0.85 (0.77, 0.93) | 0.86 (0.78, 0.94) |
| **Overall**  **(n=10,159)** | 0.84 (0.81, 0.87) | 0.85 (0.82, 0.88) | 0.87 (0.84, 0.89) |
| **Subset of cases referred for diarrhea**  **(n=3578)** | 0.78 (0.74, 0.82) | 0.79 (0.75, 0.83) | 0.80 (0.77, 0.83) |

***** No hospitalization for diarrhea occurred in Brazil
