# Supplementary material for: A Comparison of Diarrheal Severity Scores in the MAL-ED Multisite Community-Based Cohort Study
Source: J Pediatr Gastroenterol Nutr. 2016 Oct 24;63(5):466–73. doi: 10.1097/MPG.0000000000001286 (PMC5084640; doi:10.1097/MPG.0000000000001286)
Supplement: Supplemental Digital Content [file jpga-63-466-s002.docx]

**Appendix 1: Comparison to MSD as defined by healthcare worker diagnosed dysentery**

Ina subset of sites, text field in the REF form describing the HCW diagnosis also allowed for a HCW-based diagnosis of dysentery to be extracted by searching all text fields for the terms such as “dysentery” and “invasive diarrhea”, and then checking each text field manually to confirm the diagnosis. Search terms are listed in **Table A.1**.This information was used to investigate the association between the maternal-report derived diarrhea severity scores.

In three sites (BGD, NEP, PEL), a substantial number of instances of healthcare-worker defined dysentery were positively identified (BGD=229, NEP=68, PEL=78). In INV 13 instances of HCW-defined dysentery were identified and in TZH 2 cases were identified.

IN BGD, NEP, and PEL, maternally-reported blood in stool was strongly associated with the odds of receiving a HCW diagnosis of dysentery (OR=39.6, p-value<0.001, Cohen’s kappa=0.35, p-value<0.001), as was maternally-reported fever (OR=1.45, p-value=0.015). Maternally-reported vomiting was associated with a decreased odds of HCW-diagnosed dysentery (OR=0.69, p-value=0.34). Other symptoms were not statistically significantly associated with the odds of HCW-diagnosed dysentery. Among episodes that were first referred for diarrhea, the AUC of HCW-diagnosed dehydration and/or dysentery in predicting hospitalization was 0.64 (0.62, 0.66).

Among those episodes that were referred for diarrhea, mean severity scores were similar between episodes diagnosed as dysenteric, and those that were not (Clark score 4.3 versus 4.3, p=0.3373, MAL-ED score 4.1 versus 4.0, p=0.2608, CODA score 3.2 versus 3.1, p=0.2530). When MSD was defined based on HCW-defined dehydrating diarrhea, HCW-defined dysentery, or hospitalization for diarrhea, the AUCs overall were 0.64 (Clark), 0.65 (MAL-ED), and 0.63 (CODA) (among those episodes referred for diarrhea), which was lower than the AUC when MSD was defined using the mother’s report of dysentery instead. The best agreement was in the PEL site (AUC of 0.74, 0.78, and 0.77 for Clark, MAL-ED and CODA scores, respectively) and both BGD and NEB were lower (AUCs of 0.54-0.61 for all).

**Table A2.1**

HCW diagnosed dysentery was assumed whenever any of the following terms appeared in the REFDOTH (“REFerral – Diagnosis – OTHer”) text field of the referral form.

"BLOOD IN STOOL", "BLOODY STOOL", “EDA CON SANGRE”

"DYSENTERY", "DYSENTRY", "DISENTERICA", "DISENTERIA", "DISENTERICA", "DISINTERICA", "DISENTORICA", DISENFERICA"

"INVASIB DIARRHOEA", "INVASIB DIEARRHEA", "INVASIM DIARRHOEA", "INVASIVE DIARRHOEA", "IMASIVE DIARRHOEA", "INVASIBLE DIARRHOEA", "NIVASIVE DIARRHOEA", "INVASIVE DIARRHOES", "INVASIVE DIARREHOEA", "INVAXIVE DIARRHEA", "INVESIB DIARRHEA", "INVASIVE DOARRHOEA", "INVASIVE DIARRHOEA", "INVASIVE DIARRHEA", "INVASSIVE DIARRHOEA

The following strings were excluded:

"NO DYSENTERY", "NO DISENTERICA", "NO DISENTERY" "NI DISENTERICA"
