# Supplementary material for: A Comparison of Diarrheal Severity Scores in the MAL-ED Multisite Community-Based Cohort Study
Source: J Pediatr Gastroenterol Nutr. 2016 Oct 24;63(5):466–73. doi: 10.1097/MPG.0000000000001286 (PMC5084640; doi:10.1097/MPG.0000000000001286)
Supplement: Supplemental Digital Content [file jpga-63-466-s003.docx]

**MAL-ED Investigators and Institutional Affiliations**

Maribel Paredes Olotegui^1^

Cesar Banda Chavez^1^

DixnerRengifo Trigoso^1^

Julian Torres Flores^1^

Angel Orbe Vasquez^1^

Silvia Rengifo Pinedo^1^

Angel Mendez Acosta^1^

Imran Ahmed^2^

Didar Alam^2^

Asad Ali^2^

Zulfiqar A Bhutta^2^

Shahida Qureshi^2^

Muneera Rasheed^2^

Sajid Soofi^2^

Ali Turab^2^

Aisha K Yousafzai^2^

Anita KM Zaidi^2^

Ladaporn Bodhidatta^3^

Carl J Mason^3^

Sudhir Babji^4^

Anuradha Bose^4^

M. Steffi Jennifer^4^

Sushil John^4^

Gagandeep Kang^4^

Shiny Kaki^4^

Beena Koshy^4^

Jayaprakash Muliyil^4^

Mohan Venkata Raghava^4^

Anup Ramachandran^4^

Anuradha Rose^4^

Srujan L. Sharma^4^

Rahul J. Thomas^4^

William Pan^5,6^

Ramya Ambikapathi^6^

Danny Carreon^6^

Vivek Charu^6^

Leyfou Dabo^6^

Viyada Doan^6^

Jhanelle Graham^6^

Christel Hoest^6^

Stacey Knobler^6^

Dennis Lang^6,7^

Benjamin McCormick^6^

Monica McGrath^6^

Mark Miller^6^

Archana Mohale^6^

Gaurvika Nayyar^6^

Stephanie Psaki^6^

Zeba Rasmussen^6^

Stephanie A Richard^6^

Jessica C Seidman^6^

Vivian Wang^6^

Rebecca Blank^7^

Michael Gottlieb^7^

Karen H Tountas^7^

Caroline Amour^8^

Estomih Mduma^8^

BuligaMujaga Swema^8^

Ladislaus Yarrot^8^

Rosemary Nshama^8^

Tahmeed Ahmed^9^

AM Shamsir Ahmed^9^

Fahmida Tofail ^9^

Rashidul Haque^9^

Iqbal Hossain^9^

Munirul Islam^9^

Mustafa Mahfuz^9^

Dinesh Mondal^9^

Ram Krishna Chandyo^10^

Prakash Sunder Shrestha^10^

Rita Shrestha^10^

Manjeswori Ulak^10^

Robert Black^11^

Laura Caulfield^11^

William Checkley^11,6^

Ping Chen^11,6^

Margaret Kosek^11^

Gwenyth Lee^11^

Pablo Peñataro Yori^11^

Laura E. Murray-Kolb^12^

Barbara Schaefer^12,6^

Laura Pendergast^13^

Cláudia Abreu^14^

Alexandre Havt^14^

Hilda Costa^14^

Alessandra Di Moura^14^

Jose Quirino Filho^14,6^

Álvaro Leite^14^

Aldo Lima^14^

Noélia Lima^14^

Ila Lima^14^

Bruna Maciel^14^

Milena Moraes^14^

Francisco Mota^14^

Reinaldo Oriá^14^

Josiane Quetz^14^

Alberto Soares^14^

Crystal L Patil^16^

Pascal Bessong^17^

Cloupas Mahopo^17^

Angelina Maphula^17^

Cebisa Nesamvuni^17^

Emanuel Nyathi^17^

Amidou Samie^17^

Leah Barrett^18^

Jean Gratz^18^

Richard Guerrant^18^

Eric Houpt^18^

William Petri^18^

Rebecca Scharf^18^

James Platts-Mills^18^

Binob Shrestha^19^

Sanjaya Kumar Shrestha^19^ Tor Strand^19,15^

Erling Svensen^20,8^

**Institutions**

^1^A.B. PRISMA, Iquitos, Peru

^2^Aga Khan University, NausheroFeroze, Pakistan

^3^Armed Forces Research Institute of Medical Sciences, Bangkok, Thailand

^4^Christian Medical College, Vellore, India

^5^Duke University, Durham, NC, USA

^6^Fogarty International Center/National Institutes of Health, Bethesda, MD, USA

^7^Foundation for the NIH, Bethesda, MD, USA

^8^Haydom Lutheran Hospital, Haydom, Tanzania

^9^icddr,b, Dhaka, Bangladesh

^10^Institute of Medicine, Tribhuvan University, Kathmandu, Nepal

^11^Johns Hopkins University, Baltimore, MD, USA

^12^The Pennsylvania State University, University Park, PA, USA

^13^Temple University, Philadelphia, PA, USA

^14^Universidade Federal doCeara, Fortaleza, Brazil

^15^University of Bergen, Norway

^16^University of Illinois at Chicago, IL, USA

^17^University of Venda, Thohoyandou, South Africa

^18^University of Virginia, Charlottesville, VA, USA

^19^Walter Reed/AFRIMS Research Unit, Kathmandu, Nepal

^20^Haukeland University Hospital, Bergen, Norway
